# Repurposing Fragile X Drugs to Inhibit SARS-CoV-2 Viral Reproduction

**DOI:** 10.3389/fcell.2020.00856

**Published:** 2020-08-26

**Authors:** Cara J. Westmark, Maki Kiso, Peter Halfmann, Pamela R. Westmark, Yoshihiro Kawaoka

**Affiliations:** ^1^Department of Neurology, University of Wisconsin–Madison, Madison, WI, United States; ^2^Division of Virology, Department of Microbiology and Immunology, Institute of Medical Science, The University of Tokyo, Tokyo, Japan; ^3^Department of Pathobiological Sciences, School of Veterinary Medicine, Influenza Research Institute, University of Wisconsin–Madison, Madison, WI, United States; ^4^Department of Special Pathogens, International Research Center for Infectious Diseases, Institute of Medical Science, The University of Tokyo, Tokyo, Japan

**Keywords:** coronavirus, COVID-19, fragile X mental retardation protein, metabotropic glutamate receptor 5, protein synthesis

## Abstract

The COVID-19 pandemic is a global health crisis that requires the application of interdisciplinary research to address numerous knowledge gaps including molecular strategies to prevent viral reproduction in affected individuals. In response to the Frontiers Research Topic, “*Coronavirus disease (COVID-19): Pathophysiology, Epidemiology, Clinical Management, and Public Health Response*,” this Hypothesis article proposes a novel therapeutic strategy to repurpose metabotropic glutamate 5 receptor (mGluR_5_) inhibitors to interfere with viral hijacking of the host protein synthesis machinery. We review pertinent background on SARS-CoV-2, fragile X syndrome (FXS) and metabotropic glutamate receptor 5 (mGluR_5_) and provide a mechanistic-based hypothesis and preliminary data to support testing mGluR_5_ inhibitors in COVID-19 research.

## Introduction

In December of 2019, an outbreak of respiratory disease began in Wuhan, China. The causative agent was a novel betacoronavirus of the same subgenus as severe acute respiratory syndrome (SARS) coronavirus (CoV) and was named SARS-CoV-2, a.k.a. novel CoV (nCoV-2019), which causes the disease coronavirus-19 (COVID-19) (Zhu et al., 2020). COVID-19 quickly spread worldwide with clinical manifestations ranging from mild respiratory symptoms to severe pneumonia and fatality. We submit this Hypothesis paper as an interdisciplinary research approach supporting a molecular-based therapeutic strategy to reduce virus reproduction in individuals infected with SARS-CoV-2. Specifically, we provide the conceptional framework to support testing metabotropic glutamate receptor 5 (mGluR_5_) inhibitors to attenuate virus reproduction. Inhibitors of mGluR_5_ have been extensively studied in the neurodevelopmental disorder fragile X syndrome (FXS) as well as other psychiatric disorders (Gravius et al., 2010; Michalon et al., 2012; Scharf et al., 2015; Berry-Kravis et al., 2017). Inhibition of mGluR_5_ represses exaggerated protein synthesis that occurs in the absence of the RNA binding protein fragile X mental retardation protein (FMRP) (Bear et al., 2004; Osterweil et al., 2010). Work by Soto-Acosta and colleagues in 2018 demonstrates that FMRP represses Zika virus (ZIKV) infection by blocking viral RNA translation (Soto-Acosta et al., 2018). Thus, we propose that mGluR_5_ inhibitors may be a viable therapeutic strategy to interfere with the ability of SARS-CoV-2 to hijack the host cell translational machinery.

## Conceptual Framework

### SARS-CoV-2

SARS-CoV-2 is a betacoronavirus of the same family as Middle East Respiratory Syndrome (MERS-CoV) and SARS-CoV (Zhu et al., 2020). Betacoronaviruses are enveloped, non-segmented, positive-sense, single-stranded RNA [ssRNA(+)] viruses of zoonotic origin that replicate in the host cell cytoplasm and induce fever and respiratory symptoms. Infection starts by attachment of the receptor binding domain of the spike protein to host cell receptors, which mediates endocytosis of the virus into the cell and release of the ssRNA(+) viral genome into the cytoplasm. The SARS-CoV-2 and SARS-CoV receptor binding domains of spike protein share angiotensin-converting enzyme 2 (ACE2) as the host cell receptor (Li et al., 2003; Li, 2015; Wan et al., 2020). Protein-protein docking experiments and molecular dynamics (MD) simulations indicate that SARS-CoV-2 binds to ACE2 with a higher affinity than SARS-CoV, but the interaction is more temperature sensitive (He et al., 2020). These data may explain why SARS-CoV-2 is more infectious than SARS-CoV and suggest that the infection ability of SARS-CoV-2 will decline faster. Once inside the host cell, the coronavirus ssRNA(+) viral genome is used as a template to synthesize viral proteins. The viral RNA (vRNA) appears to evade the host immune system by mimicking cellular mRNA. When a critical mass of new virions are manufactured, they bud at membranes of the endoplasmic reticulum and/or Golgi apparatus and are released by exocytosis.

Regarding post-transcriptional regulation of CoV RNAs, the ∼30 kb viral RNA includes a 5′-leader sequence, 5′-untranslated region (UTR), coding sequences for viral proteins, 3′-UTR and poly(A) tail (Fehr and Perlman, 2015). The 5′-proximal two-thirds of the RNA encodes the replicase mRNA that contains 2 open reading frames, ORF1a and ORF1b. The 3′ third of CoV RNA encodes structural and accessory proteins. First, the viral RNA is translated to generate viral proteins required for transcription. Translation of ORF1a yields polyprotein 1a (pp1a), and a −1 ribosomal frame shift translates ORB1b to yield pp1ab. Together these polyproteins are processed into 16 non-structural proteins, which drive viral RNA replication and subgenomic mRNA (sgmRNA) synthesis. Specifically, the ssRNA(+) viral genome is a template for synthesis of double stranded (dsRNA), which is transcribed, thereby providing new ssRNA(+) viral genomes as well as nested sets of subgenomic mRNAs (sgmRNA) that encode structural proteins. The sgmRNA, like the RNA genome, can function as a template for negative strand RNA synthesis (Wu and Brian, 2010).

Of relevance to our hypothesis, the virus needs to commandeer the host cell protein synthesis machinery in order to propagate. Protein synthesis is dependent on the interactions between *trans* factors (RNA binding proteins, RBP) and *cis*-regulatory RNA elements. Specifically, *cis*-regulatory elements in CoV RNA need to interact with host cell RBP to translate viral mRNA. The detailed RNA-protein interactions that mediate the post-transcriptional gene regulation of SARS-CoV-2 RNA remain to be determined; however, we can predict pivotal players based on current literature. We hypothesize that FMRP, which functions as a protein synthesis inhibitor, is a pivotal molecular player and that drugs under investigation to reduce exaggerated protein synthesis in FXS may be applicable to attenuate viral protein synthesis.

### Fragile X Syndrome, FMRP and ZIKV Subgenomic RNA

Fragile X syndrome is a neurodevelopmental disorder clinically characterized by low IQ, autistic-like behaviors and seizures (Hagerman and Hagerman, 2002). FXS results from a mutation in the *FMR1* gene on the X chromosome, which is associated with transcriptional silencing of the *FMR1* promoter and loss of expression of FMRP (Verkerk et al., 1991). FMRP is a mRNA binding protein that associates with polysomes or non-translating ribonucleoprotein (RNP) particles and is involved in the transport, localization and translational repression of hundreds of mRNAs (Feng et al., 1997a, b; Weiler et al., 1997; Brown et al., 2001; Darnell et al., 2001; Laggerbauer et al., 2001; Li et al., 2001; Mazroui et al., 2002; Miyashiro et al., 2003; Bagni and Greenough, 2005).

In 2018, Soto-Acosta and colleagues published an article, “*Fragile X mental retardation protein is a Zika virus restriction factor that is antagonized by subgenomic flaviviral RNA*” (Soto-Acosta et al., 2018). Briefly, FMRP is a host factor that inhibits ZIKV translation by binding to the 3′-UTR of ZIKV subgenomic flavivirus RNAs (sfRNAs). The flavivirus life cycle is completely dependent on the cytoplasmic fate of one RNA species, namely the genomic vRNA, whose replication occurs entirely in the cytoplasm and does not generate any DNA intermediates. To create an environment favorable to infection, flaviviruses have evolved mechanisms to dampen antiviral processes, notably through the production of specific vRNA degradation products termed subgenomic flavivirus RNA (sfRNA). sfRNAs are RNAs produced by the viral replication machinery but do not contribute to synthesizing viral proteins and are non-infectious (Mazeaud et al., 2018; Berthoux, 2020). These sfRNAs bind to and inhibit the activity of host proteins that would normally block virus multiplication. FMRP is one of those proteins that binds to Zika sfRNA and inhibits the production of viral proteins. In the absence of FMRP, both the rate of infection and translation of viral protein increase per cell; i.e., knockdown of FMRP increases the infection rate ∼50–80%. Soto-Acosta and colleagues hypothesized that because FMRP is a known repressor of cellular mRNA translation, that translation of ZIKV is inhibited by FMRP early after infection thus reducing ZIKV infection, but as infection progresses, sfRNA antagonizes FMRP function leading to increased expression of FMRP target genes. Overall, the findings by Soto-Acosta et al. strongly suggest that FMRP plays a pivotal role in ZIKV infection and pathogenesis through regulation of protein synthesis.

Over two decades of studies elucidating the function of FMRP demonstrate that this RBP regulates cellular protein synthesis through multiple mechanisms including stalling polyribosomes, associating with miRNA and mRNA ribonucleoprotein complexes, and regulating the formation of RNA granules including processing (P)-bodies and stress granules (Lai et al., 2020). FMRP interacts with at least 180 other proteins of which 30% are ribosomal assembly factors (Taha et al., 2020). Thus, lack of expression of this pivotal translation regulator in FXS, or sequestration of FMRP by viral RNA, is expected to have large effects on cellular protein synthesis. Negative allosteric modulation of mGluR_5_ rescues elevated protein synthesis in mouse models of FXS and tuberous sclerosis (Michalon et al., 2012; Kelly et al., 2018).

### Fragile X Syndrome, FMRP and the Immune System

Interestingly, FXS is associated with dysregulation of the immune system, with an over-representation of infectious diseases and an under-representation of autoimmune disorders (Yu et al., 2020). Patients with FXS exhibit a significantly altered cytokine profile compared to controls. Plasma protein levels of the cytokine IL-1α are elevated and numerous serum chemokines are reduced (Ashwood et al., 2010; Van Dijck et al., 2020). The reduced levels of pro-inflammatory chemokines may indicate that the FXS immune system has a decreased capacity to respond to infection. Of importance, activation of peripheral blood mononuclear cells (PBMC) from patients with FXS with a group 1 mGluR agonist results in increased production of pro-inflammatory cytokines compared to PBMC from control subjects (Careaga et al., 2014a). The increase in cytokine production can be blocked with an mGluR_5_ antagonist. In addition to cytokine profiles, patients with FXS have an increased propensity to exhibit elevated serum anti-neuronal antibodies (43% of males) (Lisik et al., 2015). Non-human FXS models also exhibit dysregulation of the immune system. *Drosophila melanogaster Fmr1* mutants are defective in controlling bacterial infection by *S. pneumoniae* or *S. marcescens* compared to wild type flies (O’Connor et al., 2017). Peripheral immune system function appears normal in *Fmr1*^*KO*^ mice, but the mutant mice exhibit elevated hippocampal IL-1β and IL-6 mRNA compared to wild type controls at 4 h post-stimulation with lipopolysaccharide (Yuskaitis et al., 2010; Hodges et al., 2020). In contrast to full-mutation FXS, women carriers with the FXS premutation have an increased comorbidity of immune-mediated disorders and decreased cytokine production of GM-CSF and IL-12 (p40) compared to controls (Winarni et al., 2012; Careaga et al., 2014b; Jalnapurkar et al., 2015). Overall, these studies suggest that altered FMRP levels are associated with aberrant immune system function. It remains to be determined if persons with FXS are more susceptible to infection by SARS-CoV-2 and other viruses, and conversely, if the *FMR1* premutation is protective against viral infection.

### SARS-CoV-2 Negative Sense RNA Contains a Canonical FMRP Binding Site

**FIGURE 1:**
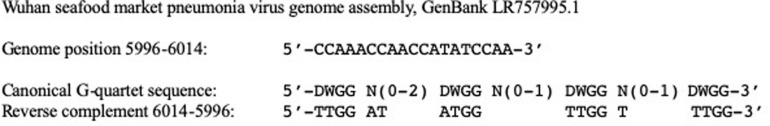
SARS-Cov-2 negative sense RNA contains a canonical G-quartet FMRP binding site. FMRP binds to target RNAs via G-quartet *cis*-regulatory elements through the consensus sequence 5′-DWGG N_(0–2)_ DWGG N_(0–1)_ DWGG N_(0–1)_ DWGG-3′ where D = any nucleotide except C and W = A or U. The whole genome sequence of the Wuhan seafood market pneumonia virus genome assembly (GenBank LR757995.1) contains a canonical G-quartet sequence at nucleotides 6014-5996 of negative sense of SARS-CoV-2 RNA. The corresponding negative sense sequence is: 5′-TTGG-AT-ATGG-TTGG-T-TTGG-3′.

Fragile X mental retardation protein binds to hundreds of cellular target mRNAs and predominantly functions to reversibly stall ribosomal translocation of messages (Darnell et al., 2011). It is of interest to determine if FMRP is a host cell factor that binds to SARS-CoV-2 genomic RNA or sgRNA as part of a regulatory mechanism involved in SARS-CoV-2 mRNA translation. FMRP binds to target RNAs via G-quartet *cis*-regulatory elements through the consensus sequence 5′-DWGG N_(0–2)_ DWGG N_(0–1)_ DWGG N_(0–1)_ DWGG-3′ where D = A, G or U and W = A or U (Darnell et al., 2001). Based on sequence analysis of the whole genome of the Wuhan seafood market pneumonia virus genome assembly (GenBank LR757995.1), we predict that FMRP binds to negative sense of SARS-CoV-2 RNA. Specifically, there is a canonical G-quartet sequence from nucleotides 6014-5996 (Figure 1). FMRP also binds to target RNA through kissing complex *ci*s-elements with the consensus site 5′-GGGCKAAGGARK……. KAGCGRCUGG-3′ where K = G or U and R = G or A (Darnell et al., 2005). We did not find any kissing complex sequences in the positive or negative sense of SARS-Cov-2 RNA. We predict that binding of FMRP to negative sense of SARS-CoV-2 RNA sequesters FMRP such that it cannot act as a translational brake for vRNA synthesis, similar to the role sfRNA plays in antagonizing FMRP function in ZIKV.

### Molecular Modeling Predicts That FMRP Binds to SARS-CoV-2 Positive and Negative Sense RNAs

Understanding how SARS-CoV-2 interferes with RNA-related posttranscriptional processes could identify novel therapies (Maranon et al., 2020). We utilized the catRAPID algorithm^1^ to predict RNA/protein interactions relevant to SARS-CoV-2 RNA (GenBank LR757995.1). This algorithm identifies potential interactions between protein and RNA molecules by combining the contributions of secondary structure, hydrogen binding and van der Waal’s forces to generate an interaction profile (Bellucci et al., 2011; Agostini et al., 2013; Cirillo et al., 2013). First, we utilized catRAPID omics to compute which RBP are predicted to bind to positive and negative sense SARS-CoV-2 RNAs. Top-ranked RBP included several splicing and heterogeneous nuclear ribonucleoproteins (Table 1). FMRP exhibited an average interaction strength of 0.30 (range 0–0.95) with an average star value of 2.58 (range 2.35–2.75) for positive sense SARS-CoV-2 RNA based on 242 predicted interactions where 57 of those interactions had an intensity ≥0.5, which is indicative of high specificity for the interaction. FMRP exhibited an average interaction strength of 0.27 (range 0–0.99) with an average star value of 2.54 (range 2.34–2.74) for negative sense SARS-CoV-2 RNA based on 42 predicted interactions where 8 interactions had an intensity ≥0.5. The interaction strength (enrichment with respect to random interactions) was computed using a reference set of 100 random protein and 100 random RNA sequences having the same lengths as the molecules under investigation, and the star rating system is a score representing the sum of the catRAPID normalized propensity, the presence of RNA/DNA binding domains and disordered regions, and the presence of known RNA-binding motifs with the range of 0–3 (Agostini et al., 2013; Cirillo et al., 2013).

**TABLE 1:** Top catRAPID hits for RBP that bind to SARS-CoV-2 RNA.

**Positive Strand**	
CSTF2	Cleavage stimulation factor subunit 2
ESRP2	Epithelial splicing regulatory protein 2
FUS	RNA-binding protein FUS
SRSF3	Serine/arginine-rich splicing factor 3
SRSF4	Serine/arginine-rich splicing factor 4
SRSF5	Serine/arginine-rich splicing factor 5
SRS10	Serine/arginine-rich splicing factor 10
SSB	Lupus La protein
YBX1	Y-box-binding protein 1
**Negative Strand**	
ESRP2	Epithelial splicing regulatory protein 2
HNRNPF	Heterogeneous nuclear ribonucleoprotein F
HNRNPH1	Heterogeneous nuclear ribonucleoprotein H
HNRNPH2	Heterogeneous nuclear ribonucleoprotein H2
QK1	Protein quaking
SFPQ	Splicing factor, proline- and glutamine-rich
SRSF3	Serine/arginine-rich splicing factor 3
SRSF5	Serine/arginine-rich splicing factor 5
TIA1	Nucleolysin TIA-1 isoform p40
TRA2B	Transformer-2 protein homolog beta

Second, we utilized catRAPID Global Score with uniform fragmentation to predict FMRP (GenBank AAH86957.1)/SARS-CoV-2 interactions. The Global Score predicts the overall interaction ability of a protein-RNA pair based on an algorithm trained on data generated by photoactivatable ribonucleoside-enhanced, high-throughput sequencing of RNA isolated by crosslinking and immunoprecipitation (PAR/HITS-CLIP) (Hafner et al., 2010). The Global Scores were 0.97 for positive sense and 0.84 for negative sense SARS-CoV-2 RNA and FMRP. The top 20 predicted interaction sites were between nucleotides 11,365–12,560 for both positive and negative sense SARS-CoV-2 RNA (interaction propensity range 279–417). Additional RNA fragments with the highest interaction propensities are listed in Table 2. The fragment of FMRP with the highest binding activity for both positive and negative sense SARS-CoV-2 RNA encompassed amino acids 311–362, which partially overlaps with a known KH RNA binding domain in FMRP (amino acids 283–325) (Siomi et al., 1994). Other FMRP protein regions with high predicted binding affinity for nucleotides 11,365–12,560 for both positive and negative sense SARS-CoV-2 RNA overlapped or partially overlapped with known Agenet (63–120) KH1 (221–280), KH2 (283–325), C-terminal (C1, 399–526), and C2 (504–586) domains as well as intervening FMRP protein sequences.

**TABLE 2:** Top catRAPID hits of SARS-CoV-2 that bind to FMRP.

Fragment	Interaction Propensity
**Positive Strand**	
11365–12560	416.94
7783–8978	274.98
1216–2411	267.13
3007–4002	265.21
3604–4799	264.88
4798–5993	263.82
8380–9575	263.65
7186–8381	262.81
4201–5396	256.57
2410–3605	253.78
**Negative Strand**	
11365–12560	413.11
7186–8381	272.08
4798–5993	268.10
7783–8978	267.37
10171–11366	261.15
5992–7187	259.99
5395–6590	257.13
9574–10769	255.20
8380–9575	254.77
4201–5396	254.30

**FIGURE 2:**
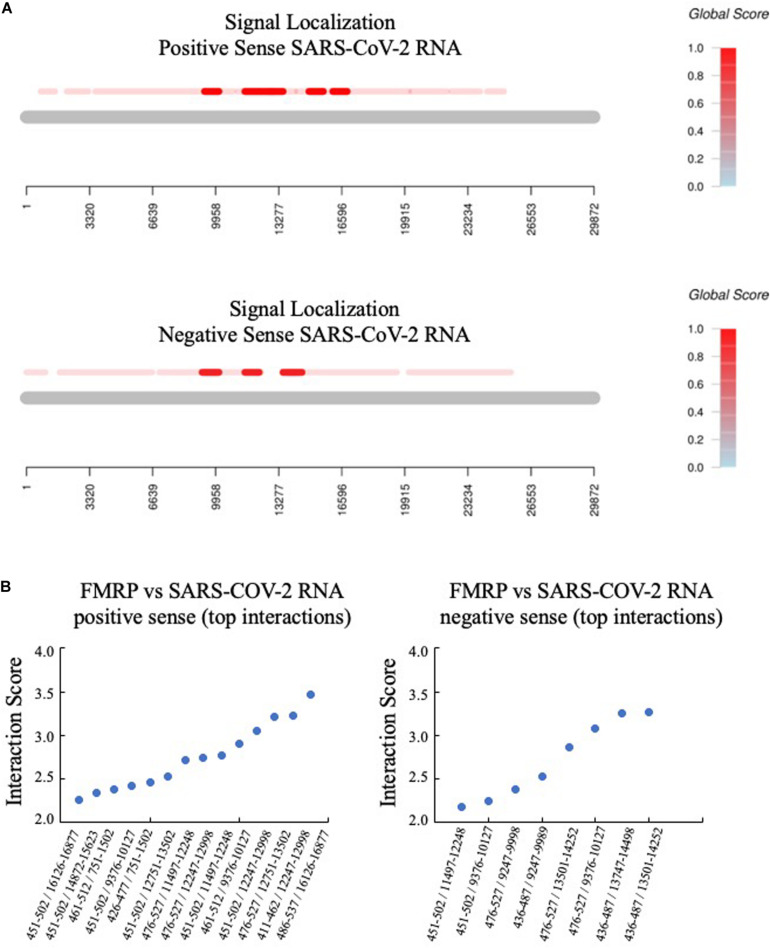
FMRP is predicted to bind to multiple regions of SARS-CoV-2 RNA. **(A)** The catRAPID Global Score algorithm with uniform fragmentation and signal localization predicts multiple FMRP binding sites in both positive and negative sense SARS-CoV-2 RNA, with the strongest predicted binding in the region of nucleotides 9,000–17,000 (positive sense) and nucleotides 9,000–15,000 (negative sense). **(B)** The top specific interactions are graphed by protein/RNA sequence positions (*x*-axis) versus interaction score (*y*-axis). Protein and RNA coordinates are reported relative to the NCBI database.

Third, the catRAPID signal localization algorithm predicted the top interactions for positive and negative sense SARS-CoV-2 RNA (Figure 2). The protein region of FMRP implicated in binding overlapped with the C1 region, which is an arginine-glycine-rich (RG-rich) region that participates in non-specific RNA binding (Adinolfi et al., 1999).

And fourth, we utilized catRAPID Global Score with weighted fragmentation to predict FMRP/SARS-CoV-2 interactions. The fragmentation weighted option generates interaction predictions using intact RBP (FMRP) and fragments of the RNA (100–200 nucleotides of positive or negative SARS-CoV-2 RNA), and is useful for the study of RNAs, which are larger than 1,000 nucleotides. FMRP and SARS-CoV-2 RNA are predicted to interact with propensities of 0.93 (positive sense) and 0.87 (negative sense). The top predicted interaction sites for positive and negative sense SARS-COV-2 are provided in the Supplementary Figure S1. Of note, 8 negative sense SARS-CoV-2 RNA fragments spanned the putative G-quartet region and exhibited an average interaction propensity of 5.8 ± 1.2 with FMRP. For comparison of interaction propensities, FMRP interacts with human mRNAs including *CAMK2A* (BC040457.1), *PSD-95* (U83192.1) and *APP* (BC065529.1) with global scores of 0.54, 0.68 and 0.71, respectively, using the weighted algorithm.

Overall, these molecular modeling studies indicate an overwhelming plentitude of potential interactions between FMRP and SARS-CoV-2 RNA, which remain to be experimentally validated. FMRP is predicted to bind along 25 kB of the 29.8 kB length of positive and negative sense SARS-CoV-2 RNAs such that the RNA could act as a sink for FMRP and other RBP and prevent their normal function. The predicted interaction propensities of FMRP with SARS-CoV-2 positive and negative sense RNAs are stronger than known FMRP target mRNAs.

### Repurposing mGluR_5_ Inhibitors for Treatment of COVID-19

The leading drug target to date for FXS is the glutamate-activated, G-protein-coupled receptor mGluR_5_, which signals through FMRP (Bear et al., 2004; Stoppel et al., 2017). The mGluRs contain a large extracellular amino terminal domain, a heptahelical transmembrane region, and an intracellular carboxy terminal domain. Negative allosteric modulators (NAMs) of mGluR_5_ bind to the transmembrane heptahelical domain. These drugs are potent, non-competitive, selective and systematically active allosteric antagonists that are under study for a range of indications including anxiety, epilepsy, pain, depression, Parkinson’s disease, gastroesophageal reflux disease, FXS, autism, and addiction (Westmark, 2014). There has been a concerted effort to repurpose mGluR_5_ NAMs for the treatment of FXS where these drugs rescue disease phenotypes in multiple preclinical models and have been safely tested in clinical trials (Gravius et al., 2010; Michalon et al., 2012; Scharf et al., 2015; Berry-Kravis et al., 2017). Although mGluR_5_ expression is enriched in brain tissue, the receptor is ubiquitously expressed in the body including the lungs^2^. We hypothesize that mGluR_5_ NAMs could be a prophylactic treatment to slow viral protein synthesis in patients infected with SARS-CoV-2.

Treatment of COVID-19 will likely require a therapeutic cocktail approach. Lead candidate drugs have been reviewed and include angiotensin receptor blockers, statins, remdesivir, chloroquine, hydroxychloroquine, lopinavir-ritonavir and interferon-beta (Kupferschmidt and Cohen, 2020). Angiotensin receptor blockers and statins upregulate ACE2, the SARS-CoV-2 host receptor, and are expected to increase the host response to infection allowing the patient to recover on their own (Fedson et al., 2020). Remdesivir shuts down viral replication by inhibiting viral RNA polymerase and has been shown to inhibit both the SARS and MERS viruses but not Ebola. Remdesivir must be given intravenously and is expensive. Chloroquine and hydroxychloroquine decrease the acidity of cellular endosomes compartments, which are involved in the degradation of foreign material. These drugs require high doses that could cause severe toxicity and many side effects. Lopinavir-ritonavir inhibits the HIV protease and has been shown effective in marmosets infected with the MERS-CoV virus. Interferon-beta regulates inflammation. A combination of lopinavir-ritonavir with interferon-beta has lessened disease severity in marmosets with MERS-CoV but could be risky for patients with severe COVID-19 and lead to more tissue damage. Other drugs under investigation for COVID-19 include corticosteroids and baricitinib, which reduce inflammation in the treatment of rheumatoid arthritis; camostat mesylate, which inhibits a human protein involved with infection; anti-viral drugs including the influenza drug favipiravir; and additional HIV antivirals (Kupferschmidt and Cohen, 2020). An alternative therapeutic strategy is to boost immunity with plasma from convalesced COVID-19 patients or monoclonal antibodies directed at SARS-CoV-2.

We propose that inclusion of mGluR_5_ NAMs as part of a drug cocktail approach to combat COVID-19 offers the advantages of: (1) extensive preclinical research regarding its mechanism of action; (2) prior safety testing in human clinical trials of FXS; (3) numerous mGluR_5_ NAMs available from multiple pharmaceutical countries worldwide; (4) orally dosed; (5) protein target ubiquitously expressed including the lungs; (6) less expensive to produce small molecule drugs; and (7) targets a post-transcriptional gene regulatory step in viral production not addressed by other therapies under investigation. In addition, blockade of mGluR_5_ activity prevents an increase in proinflammatory cytokines and chemokines (Shah et al., 2012), which may quell the cytokine storm elicited by SARS-CoV-2 infection.

### Preclinical Testing Strategy of mGluR_5_ Inhibitors in SARS-COVID-2 Models

Proposed experiments to validate our hypothesis include: (1) gel mobility shift and co-immunoprecipitation assays to identify FMRP/SARS-Cov-2 RNA interactions, (2) *in vitro* translation assays to quantitate viral and cellular protein synthesis levels in the presence and absence of FMRP and mGluR_5_ inhibitors, (3) *in vitro* assays in SARS-CoV-2 RNA-infected cells that under- and over-express FMRP to assess protein synthesis levels and virus production with/without mGluR_5_ inhibitors, and (4) *in vivo* testing of disease outcomes in a COVID-19 animal model in response to mGluR_5_ inhibitors. An important caveat to this hypothesis is that viruses can differentially affect the host translational machinery. It will be important to test both mGluR_5_ NAMs and positive allosteric modulators (PAMs) in preclinical studies to ascertain effects on SARS-CoV-2 RNA and protein synthesis. To our knowledge, the only study testing mGluR_5_ drugs in a virus model was in a virus-induced temporal lobe epilepsy (TMEV) model where treatment with the PAM VU0360172 reduced acute seizures, while blocking mGluR_5_ did not make seizure phenotypes worse (Hanak et al., 2019).

**FIGURE 3:**
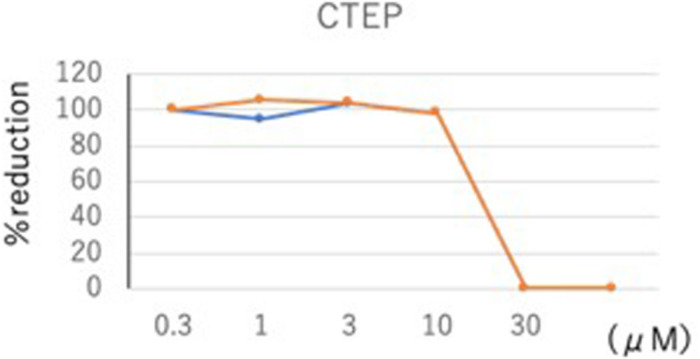
CTEP reduces SARS-CoV-2 viral plaque load. SARS-CoV-2 virus titer was assessed in response to a 100-fold concentration range (0.3–30 μM) of the mGluR_5_ inhibitor CTEP. Confluent VeroE6/TPMRSS2 cells seeded at a density of 5 × 10^5^ cells per well in 6-well plates were infected with SARS-CoV-2 [approximately 80 plaque-forming units of SARS-CoV-2/UT-NCGM02/Human/2020/Tokyo (UT-NCGM02) isolated from a mild case in Tokyo]. After 30 min infection, viral inoculum was removed and cells were washed three times to remove any unbound virus before being overlaid with MEM containing a final concentration of 5% fetal bovine serum and 1.0% methyl cellulose (Sigma-Aldrich; to allow for plaque formation) along with various concentrations of CTEP. After an incubation of 2 days, the number of plaques were counted and IC_50_ values were calculated using Graphpad Prism software (Graphpad Software, La Jolla, CA, United States). CTEP reduced virus titer with an IC_50_ of 13.1 μM.

Toward validation of our model, we tested the mGluR_5_ inhibitor 2-chloro-4-((2,5-dimethyl-1-(4-(trifluoro- methoxy)phenyl)-1H-imidazol-4-yl)ethynyl)pyridine (CTEP) in an *in vitro* SARS-CoV-2 assay (Figure 3). CTEP is a commercially available, research-grade mGluR_5_ inhibitor developed by Hoffmann-La Roche. This negative allosteric modulator of mGluR_5_ acts with nanomolar affinity and greater than 1,000-fold selectivity when tested against 103 targets (Lindemann et al., 2011). CTEP has high oral bioavailability and long duration of action in animal models with a single dose lasting 18 h. CTEP reduced viral plaque load with an IC_50_ of 13.1 μM in a VeroE6/TMPRSS2 cell assay. *In vivo* testing of CTEP in a hamster COVID-19 model is in progress.

### Clinical Feasibility

The old adage, “*feed a cold, starve a fever*,” may apply to treating COVID-19. Starving a fever is medical folklore for normalizing metabolism that is in overdrive. Metabolism is dependent on protein synthesis. Because virus translation dominates host cell translation at later time points of infection due to the high level of viral transcripts (Irigoyen et al., 2016), reducing protein synthesis after the onset of symptoms would be predicted to starve virus translation more than host cell translation leading to reduced virus production and affording the adaptive immune system more time to generate a response.

**FIGURE 4:**
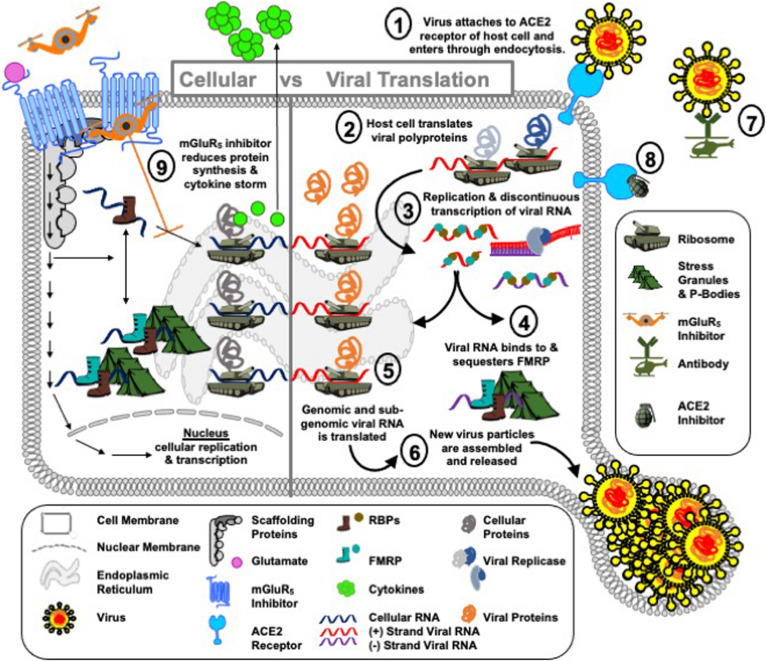
Operation mGluR_5_. COVID-19 is a global pandemic, i.e., a Blitzkrieg attack by SARS-CoV-2 on the human population. The port of attack for SARS-CoV-2 is the cell surface receptor angiotensin-converting enzyme 2 (ACE2) in the cells of the lungs ➀. Once SARS-CoV-2 lands and breaches the cell border, the virus injects positive-sense, single-stranded RNA [ssRNA(+)] into the cell cytoplasm and immediately takes hostage of the host cell protein synthesis machinery ➁ to replicate and transcribe new viral RNA ➂. This is accomplished by a swift and effective disarmament of the cell’s shock troops, RNA binding proteins (RBPs). Shock troops is a military term for infantry formations created to lead an attack. In the RNA world, RBPs bind to RNA to either degrade, localize, store or translate messages. RBPs can bind to viral as well as cellular mRNA. In the case of viral infection, viral RNA recruits cellular RBPs to translate viral proteins at ribosomes, or sequesters cellular RBPs at other cell encampments, such as stress granules and P-bodies, to block their normal cellular function. We hypothesize that FMRP is a shock troop that is sequestered by SARS-CoV-2 RNA to prevent its normal function of acting as a brake on protein synthesis ➃. In the absence of FMRP, it is predicted that the rate of viral protein synthesis ➄ and hence further infection ➅ are increased. Public surveillance policies and on-site diagnostics (i.e., equivalent to wartime communications and intelligence reports) to inform the public and health care professionals on viral spread are in place. Vaccines, which can mediate a rapid immune response to fight infection are in progress (i.e., cell airborne attack) ➆. Drugs to target ACE2, the port of infection, are identified and under study (i.e., cell naval response) ➇. What we lack in the fight against SARS-CoV-2 are drugs that support the boots on the ground, i.e., RBPs, and protect their encampments, i.e., ribosomes, stress granules and P-bodies. We propose that mGluR_5_ inhibitors ➈ are a potential drug therapy to combat viral hijack of the host translational infrastructure (i.e., the cell army) by slowing down protein synthesis to afford the innate immune system time to identify a viral infection and mediate an adaptive response as well as to afford the cell degradation machinery (i.e., cell marines) time to recruit and degrade viral proteins. It is anticipated that reduced protein synthesis could have negative consequences for the host cell as well as the virus; however, similar to chemotherapy that kills both healthy and cancer cells, this defensive strategy to delay advance of the stealth virus invader could buy time until the enemy can be eradicated by flanking troops. An additional potential benefit of mGluR_5_ inhibition is reduced cytokine production, which could attenuate the COVID-19 cytokine storm.

Inhibitors of mGluR_5_, which have been extensively studied in both preclinical research and in clinical trials, particularly as regards FXS and Parkinson’s disease (PD) (Gravius et al., 2010; Michalon et al., 2012; Scharf et al., 2015; Tison et al., 2016; Berry-Kravis et al., 2017), offer a potential repurposing strategy for COVID-19 (Figure 4). The FXS field has three decades of experience in mobilizing academic, pharmaceutical, biotechnology and clinical partners to repurpose drugs for a rare disorder through the efforts of FRAXA Research Foundation, the National Fragile X Foundation (NFXF) and other advocacy groups. There have been over 3,000 publications by the biomedical community to understand the role of FMRP in FXS and to test promising drugs with mGluR_5_ as the leading drug target. This experience could be rapidly extrapolated to COVID-19. Of most importance, multiple clinical trials have been conducted in both children and adults with FXS as well as adults with PD with minimal adverse effects.

Limitations regarding repurposing mGluR_5_ inhibitors for COVID-19 include: (1) the need for key supporting experiments regarding the mechanism, i.e., linking mGluR_5_, FMRP and viral protein production; (2) FMRP is not the only downstream target of mGluR_5_; (3) viral protein production is not exclusively regulated by FMRP and/or mGluR_5_; (4) caution is required in the interpretation of the *in vitro* virus titer data in response to CTEP as weak activity is indicated by an IC_50_ of 13.1 μM; and (5) it is unknown if an effective serum concentration can be achieved in patients and if therapeutic doses will induce adverse reactions. Nonetheless, considering the dearth of therapeutic options for COVID-19 and the established safety profile of mGluR_5_ NAMs, it is worthwhile to test clinical grade mGluR_5_ NAMs, such as AFQ056, basimglurant and dipraglurant, in *in vitro* and *in vivo* models of COVID-19.

## Conclusion

In conclusion, public surveillance and vaccine development for COVID-19 are on-going, but we have limited knowledge of SARS-CoV-2 post-transcriptional gene regulation and a dearth of therapeutic options. Thus, there is a critical need for the research community to rapidly mobilize to address these knowledge gaps related to COVID-19. In addition, viral infections will remain a serious threat even after COVID-19 passes. Viruses are constantly mutating and have the capacity to transmit between species. It is imperative to identify an arsenal of therapeutic options. Evidence-based research to support vaccine and drug development requires time and money to conduct rigorous and reproducible studies in preclinical models to support a hypothesis followed by extensive clinical trial validation. Thus, when currently available drugs can be repurposed for a rare disorder, or a global epidemic, it can greatly reduce the cost and time of drug validation. Targeting protein synthesis as part of a therapeutic arsenal may be a feasible broad-spectrum option to target viruses, which depend on, and cannot replicate without, the host cell translational machinery.

It remains to be determined if FMRP plays a role in protein synthesis in response to SARS-CoV-2 infection, and if mGluR_5_ NAMs are a viable therapeutic strategy to modulate viral protein production. From a post-transcriptional gene regulation perspective, research questions that need to be addressed include: which RBP bind to and regulate synthesis of SARS-CoV-2 genomic and subgenomic RNA? Does SARS-Cov-2 RNA sequester and thereby inactivate host cell RBP such as FMRP to promote viral RNA production? Do drugs that target RBP attenuate viral replication? How do those drugs affect the immune response? Nonetheless, mGluR_5_ NAMs have been extensively studied in non-viral models, have proven relatively safe, and may provide a rapid repurposing strategy. Similar to physical distancing and the temporary shutdown of our economy at the national level to allow public surveillance and prevent viral spread, temporary attenuation of protein synthesis at the cellular level may afford the immune system time to find and fight SARS-Cov-2.

## Data Availability Statement

All datasets presented in this study are included in the article/Supplementary Material.

## Author Contributions

CW: conceptualization and original draft preparation. CW and MK: data collection. CW, PH, PW, and YK: review and editing. All authors contributed to the article and approved the submitted version.

## Conflict of Interest

The authors declare that the research was conducted in the absence of any commercial or financial relationships that could be construed as a potential conflict of interest.

## Footnotes

1 http://s.tartaglialab.com/page/catrapid_group

2 The Human Protein Atlas, accessed 04/02/20 at https://www.proteinatlas.org/ENSG00000168959-GRM5.
