# Supplementary material for: Repurposing Fragile X Drugs to Inhibit SARS-CoV-2 Viral Reproduction
Source: Front Cell Dev Biol. 2020 Aug 26;8:856. doi: 10.3389/fcell.2020.00856 (PMC7479061; doi:10.3389/fcell.2020.00856)
Supplement: Supplementary file 1 [file Image_1.pdf]

Repurposing Fragile X Drugs to Inhibit SARS-CoV-2 Viral Reproduction

Cara J. Westmark[1*], Maki Kiso[2], Peter J. Halfmann[3], Pamela R. Westmark[1], Yoshihiro Kawaoka[2,3,4]

[1]Department of Neurology, University of Wisconsin, Madison, WI, USA
[2]Department of Virology, Department of Microbiology and Immunology, Institute of Medical Science, University of Tokyo, Tokyo, Japan
[3]Influenza Research Institute, Department of Pathobiological Sciences, School of Veterinary Medicine, University of Wisconsin, Madison, WI, USA
[4]Department of Special Pathogens, International Research Center for Infectious Diseases, Institute of Medical Science, University of Tokyo, Tokyo, Japan

*** Correspondence:**
Cara Westmark
westmark@facstaff.wisc.edu

Supplementary Data

Supplementary Figure 1: FMRP is predicted to bind to multiple regions of SARS-CoV-2 RNA. The catRAPID Global Score algorithm with weighted fragmentation predicts multiple FMRP binding sites in both positive (A) and negative (B) sense SARS-CoV-2 RNA. The top 20 interactions are displayed with RNA sequence positions on the x-axis versus interaction score on the y-axis. RNA coordinates are reported relative the NCBI database.

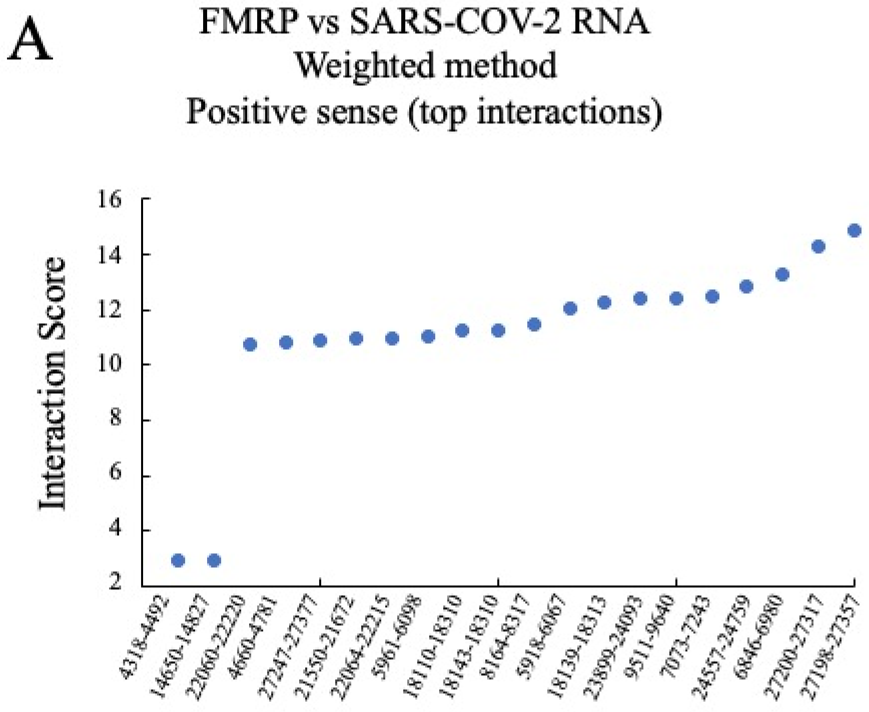
A
FMRP vs SARS-COV-2 RNA
Weighted method
Positive sense (top interactions)
Interaction Score
16
14
12
10
8
6
4
2
4318-4492
14650-14827
22060-22220
4660-4781
27247-27377
21550-21672
22064-22215
5961-6098
18110-18310
18143-18310
8164-8317
5918-6067
18139-18313
23899-24093
9511-9640
7073-7243
24557-24759
6846-6980
27200-27317
27198-27357

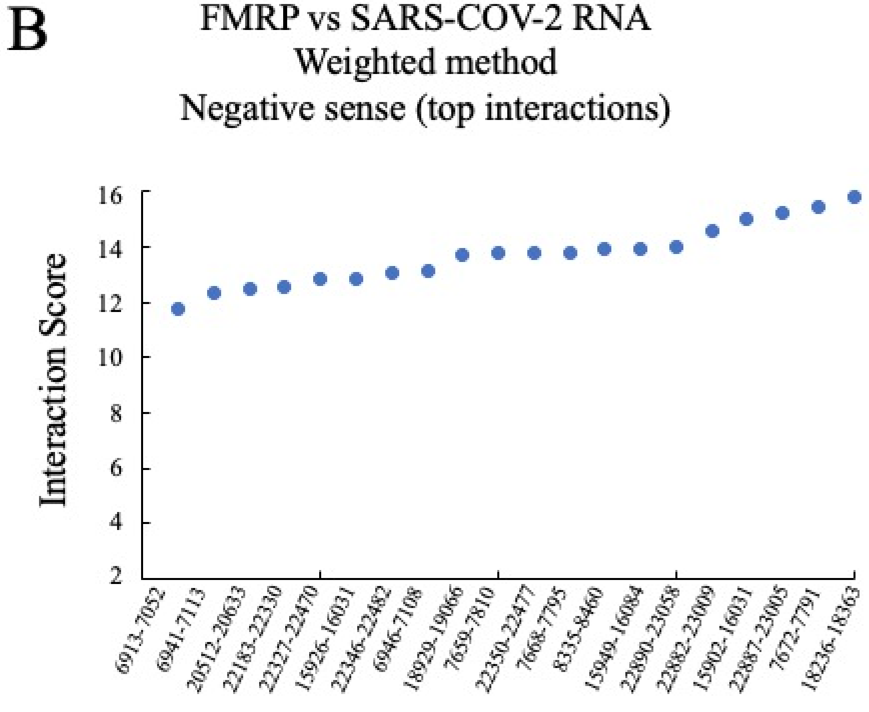
B
FMRP vs SARS-COV-2 RNA
Weighted method
Negative sense (top interactions)
Interaction Score
16
14
12
10
8
6
4
2
6913-7052
6941-7113
20512-20633
22183-22330
22327-22470
15926-16031
22346-22482
6946-7108
18929-19066
7659-7810
22350-22477
7668-7795
8335-8460
15949-16084
22890-23058
22882-23009
15902-16031
22887-23005
7672-7791
18236-18363
